# Triterpenoid CDDO-Me induces ROS generation and up-regulates cellular levels of antioxidative enzymes without induction of DSBs in human peripheral blood mononuclear cells

**DOI:** 10.1007/s00411-020-00847-w

**Published:** 2020-05-14

**Authors:** Christina Beinke, Harry Scherthan, Matthias Port, Tanja Popp, Cornelius Hermann, Stefan Eder

**Affiliations:** 1 Bundeswehr Institute of Radiobiology Affiliated to the University Ulm, Neuherbergstr. 11, 80937 Munich, Germany; 2 Institute and Outpatient Clinic for Occupational, Social and Environmental Medicine, Inner City Clinic, University Hospital of Munich (LMU), Munich, Germany

**Keywords:** Bardoxolone-methyl, CDDO-me, Antioxidant, Radioprotective activity, DNA damage, Micronucleus assay, γ-H2AX foci analysis

## Abstract

Ionizing radiation produces reactive oxygen species (ROS) leading to cellular DNA damage. Therefore, patients undergoing radiation therapy or first responders in radiological accident scenarios could both benefit from the identification of specifically acting pharmacological radiomitigators. The synthetic triterpenoid bardoxolone-methyl (CDDO-Me) has previously been shown to exert antioxidant, anti-inflammatory and anticancer activities in several cell lines, in part by enhancing the DNA damage response. In our study, we examined the effect of nanomolar concentrations of CDDO-Me in human peripheral blood mononuclear cells (PBMC). We observed increased cellular levels of the antioxidative enzymes heme oxygenase-1 (HO-1), NAD(P)H dehydrogenase (quinone1) and mitochondrial superoxide dismutase 2 by immunoblotting. Surprisingly, we found increased intracellular ROS-levels using imaging flow-cytometry. However, the radiation-induced DNA double-strand break (DSB) formation using the γ-H2AX + 53BP1 DSB focus assay and the cytokinesis-block micronucleus assay both revealed, that nanomolar CDDO-Me pre-treatment of PBMC for 2 h or 6 h ahead of X irradiation with 2 Gy did neither significantly affect γ-H2AX + 53BP1 DSB foci formation nor the frequency of micronuclei. CDDO-Me treatment also failed to alter the nuclear division index and the frequency of IR-induced PBMC apoptosis as investigated by Annexin V-labeled live-cell imaging. Our results indicate that pharmacologically increased cellular concentrations of antioxidative enzymes might not necessarily exert radiomitigating short-term effects in IR-exposed PBMC. However, the increase of antioxidative enzymes could also be a result of a defensive cellular mechanism towards elevated ROS levels.

## Introduction

Ionizing radiation (IR) induces biological effects, amongst others, via the generation of intracellular reactive oxygen or nitrogen species (ROS, RNS) such as hydroxyl radicals, which is considered to represent a major cause for post-irradiation cell damage (Azzam et al. 2012; Maier et al. 2016; Riley 1994). Both acute and chronic biological effects of IR relate to the extent of DNA damage like DNA double-strand breaks (DSB) and the fidelity of cellular repair mechanisms. While lethal consequences of IR-induced cellular DNA damage are clinically utilized for the treatment of various cancers, they simultaneously implicate limitations for radiotherapy by the impairment of the surrounding healthy tissue. Additionally, radioprotective or radiomitigative drugs for prevention and treatment of radiation-related adverse health effects, e.g. among victims after radiological incidents or astronauts with regard to future long-term space missions, are strongly needed (Bhattacharjee 2011; Suman et al. 2013; Shay et al. 2011; Durante and Cucinotta 2008). The majority of radioprotective or -mitigative molecules currently available or under development have certain shortcomings regarding efficacy, cytotoxicity, bioavailability or tolerability, and are therefore still of limited use for both, pre-clinical and clinical administration. Therefore, numerous research efforts have been made during the last decades to identify small molecules exhibiting radioprotective characteristics for normal tissues (Suman et al. 2013).

**Fig. 1:**
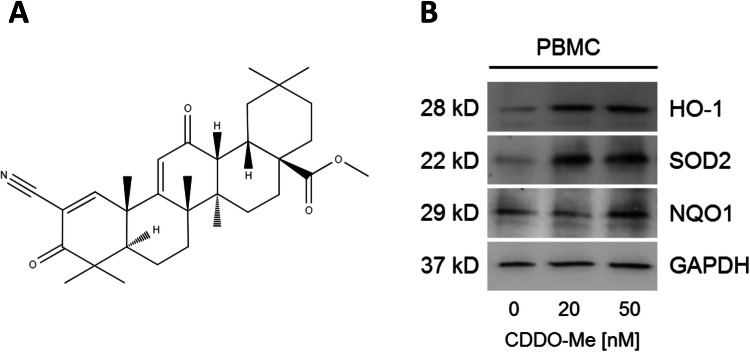
**a** Molecular structure of synthetic oleanane triterpenoid Bardoxolone-methyl or CDDO-Me (2-cyano-3,12-dioxooleane-1,9(11)-dien-28-oic acid methyl ester). **b** Incubation of PBMCs with nanomolar CDDO-Me for 6 h induced cellular accumulation of the antioxidative enzymes HO-1, NQO1 and SOD2 as shown by immunoblotting (*n* = 3). DMSO served as control (0 nM CDDO-Me)

Oleanolic acids are naturally occurring triterpenoids which have been used in traditional medicine in Asia as an anticancer and anti-inflammatory treatment (Ryu et al. 2000; Alqahtani et al. 2013). The synthetic oleanane triterpenoid bardoxolone-methyl (CDDO-Me or RTA-402, Fig. 1a) has been shown to act as a multifunctional drug in a dose-related manner, displaying cytoprotective effects by the suppression of inflammation and oxidative stress at low nanomolar concentrations, whereas increasing doses to micromolar ranges predominantly exert anti-proliferative and pro-apoptotic activities in various cancers (Liby and Sporn 2012; Sporn et al. 2011; Wang et al. 2015, 2014a, b). Recent preclinical and clinical studies addressed the potential use of CDDO-Me as an anticancer treatment and a current phase 3 clinical study is investigating its cytoprotective effects in patients with diabetic kidney disease (AYAME Study, ClinicalTrials.gov Identifier: NCT03550443) (Suh et al. 1999; Honda et al. 2000; Petronelli et al. 2009; Shanmugam et al. 2014, 2012).

Due to its widespread biological activities, CDDO-Me is capable of interacting with multiple cellular signaling cascades, like the Keap1/Nrf-2 pathway, the JAK-STAT pathway, the tumor suppressor PTEN, the mTOR complex or the NF-κB pathway (Liby and Sporn 2012; Wang et al. 2014a). The high affinity of CDDO-Me to the Kelch-Like Erythroid cell-derived protein with CNC homology-Associated Protein1 (Keap1) is regarded as a key factor for the initiation of antioxidative effects via the activation of Nrf2-signaling. Downstream binding of Nrf-2 to regulatory antioxidant response elements (ARE) finally activates the transcription of genes coding for antioxidant proteins and detoxifying enzymes, such as hemeoxygenase-1 (HO-1), NADPH dehydrogenase [quinone]-1 (NQO1) and mitochondrial manganese-dependent superoxide dismutase-2 (SOD2) (Liby and Sporn 2012). HO-1 plays a major part in the catabolism of heme, finally leading to increased amounts of the effective antioxidant bilirubin, whereas the FAD-dependent flavoprotein NQO1 classically catalyzes the reduction of a broad spectrum of quinones to hydroquinones. Finally, mitochondrial SOD2 has been shown to be essential for aerobic life by its capability to convert the physiological superoxide byproduct of the electron-transport respiratory chain to peroxide (Son et al. 2013; Dinkova-Kostova and Talalay 2010; Lebovitz et al. 1996).

Previous studies revealed the up-regulation of antioxidant Nrf2/ARE downstream targets upon administration of low nanomolar CDDO-Me in normal lung, breast and colon epithelial cells as well as in human peripheral blood mononuclear cells (PBMC), coinciding with enhanced resistance to ionizing radiation (El-Ashmawy et al. 2014; Thimmulappa et al. 2007; Kim et al. 2013; Eskiocak et al. 2010).

In this study, we examined potential effects of nanomolar CDDO-Me in PBMC from healthy donors on the homeostasis of antioxidant enzymes and focused on the implications for X-ray-induced ROS generation, induction of apoptosis and DNA DSB damage and repair.

The γ-H2AX focus assay and the cytokinesis-block micronucleus analysis (CBMN) are two established tools to assess radiation-induced cell DNA damage. The γ -H2AX + 53BP1 focus assay monitors DNA DSBs (Lassmann et al. 2010; Eberlein et al. 2015) by quantification of nuclear foci of phosphorylated (gamma-) histone H2AX. The latter is phosphorylated upon DSB induction in the chromatin surrounding a DSB site within seconds to minutes (Rogakou et al. 1998, 1999); 53BP1, a DSB sensor protein (reviewed by Mirman and de Lange 2020), also localizes to the same chromatin domains around DSBs with both proteins showing as microscopically colocalizing microscopic foci (Markova et al. 2011; Lamkowski et al. 2014; Lassmann et al. 2010). Micronuclei (MNi) are small membrane-bound DNA fragments in the cytoplasm outside the main nucleus, which originate from acentric fragments (due to failure of DSB repair or dicentric chromosomes) or whole chromosomes (e.g. dicentric chromosomes, or such with defective kinetochores) that are unable to migrate properly during the anaphase of mitosis. Radiation-induced MNi is largely the result of un- or misrepaired DSBs by the nonhomologues end-joining pathway (Fenech 2000; Vral et al. 2011). The CBMN assay is an in vitro mutagenic test system routinely used to assess the potential genotoxicity of compounds.

## Materials and methods

### Blood sampling and isolation of peripheral blood mononuclear cells (PBMC)

Venipuncture was performed with informed consent from 4 healthy volunteers (3 males, 1 female; aged between 35 and 50 years) according to the approval of the Institutional Review Board at the University of Ulm. Peripheral blood samples were drawn directly into sodium-heparin containing BD Vacutainer^®^ CPT™ tubes (Becton Dickinson, Franklin Lakes, NJ, USA) for PBMC isolation or using lithium heparinized vials to use the whole blood (CBMN) or to perform a Ficoll-paque (GE Healthcare) density centrifugation PBMC isolation (γ-H2AX + BP53 focus assay). For ROS quantification, viability assessment, apoptosis screening and CBMN PBMC isolated with the BD CPT™ system were used. PBMC purification was performed following the protocol of the manufacturer. Briefly, CPT tubes were centrifuged 20 min at 1800×*g* at room temperature to separate the PBMC layer. PBMC were collected into a fresh tube, phosphate-buffered saline (PBS; pH 7,4; without Mg^2+^/Ca^2+^) was added to a final volume of 15 mL before cells were centrifuged 15 min at 300 xg. After a second washing step, cells were suspended in 500 µL RPMI 1640 to determine the cell number and for subsequent proceedings.

### Drug pre-treatment and radiation exposure

Either PBMC or whole blood cells were incubated for 2 h or 6 h at 37 °C and 95% relative humidity in a 5% CO_2_ atmosphere applying dimethyl sulfoxide (DMSO; 0 nM CDDO-Me), or CDDO-Me (20 nM/50 nM dissolved in DMSO; Selleckchem, Houston, USA) according to the treatment protocol. Subsequently, irradiation at 2 Gy was performed at 37 °C using single doses of X-rays with mean photon energy of 100 keV. X-rays were generated using an MG325 generator/control unit and an X-ray tube type Y.TU320-D03 (equipped with a 3 mm Beryllium and 3 mm Aluminum filter) which was installed in a Maxishot SPE cabinet (Yxlon, Hamburg, Germany). The absorbed dose was measured using a UNIDOS webline type 10021 dosimeter (PTW, Freiburg, Germany). The dose-rate was approximately 1.0 Gy min^−1^ at 13 mA and 240 kV. Incubation at 37 °C in a 5% CO_2_ atmosphere has been continued immediately after irradiation according to the respective protocols.

### Western blot analysis

Western blotting of PBMC whole cell lysates was performed according to standard protocols using the XCell Sure Lock™ Mini-Cell Electrophoresis System. Equalization of protein concentrations of the whole-cell lysates was performed using the BCA Protein Assay Kit according to the manufacturer´s instructions (both from Thermo Scientific, Rockford, USA).

HRP-conjugated rabbit monoclonal anti-GAPDH was used as a loading control (conc. 1:10.000, 4 °C overnight incubation, Thermo Scientific, Rockford, USA). The primary antibodies used were rabbit monoclonal anti HO-1, mouse monoclonal anti-NQO1 and rabbit monoclonal anti-SOD2 (Cell signaling technologies, Danvers, USA; each diluted 1:1.000). Visualization of immunoreactivity was carried out by HRP-conjugated secondary antibodies (each diluted 1:10.000, DAKO A/S, Glostrup, Denmark), Super-Signal West Pico chemoluminescence (Pierce, Rockford, USA) and subsequent digital image acquisition using the myECL™ Imager system (Thermo Scientific, Westham, USA). We performed densitometry using ImageJ software, v. 1.51 (NIH, Bethesda,USA) and calculated greyscale value ratios with DMSO control after normalizing protein intensity ratios with GAPDH from the identical experiment as a reference.

### Quantification of reactive oxygen species

CPT™ isolated PBMC (using 130 IU normal heparin, 2,0 mL Ficoll; BD, Franklin Lakes, NJ, USA) were washed thrice with PBS (pH 7,4; without Mg^2+^/Ca^2+^), suspended in 10 mL serum and diluted with RPMI 1640 to a total volume of 50 mL. Afterwards, PMBCs were pre-incubated with CDDO-Me (20 nM, 50 nM) or DMSO as solvent control (5 ppm DMSO in all samples). After 5.5 h of incubation, a 20 mM 2′,7′-dichlorodihydrofluorescein diacetate (H_2_DCFDA) stock in DMSO was added to yield a 20 µM H_2_DCFDA solution and incubated for an additional 30 min. Afterwards, the PBMC were centrifuged for 10 min using 300 g with a low break, suspended in 200 µL of PBS and irradiated at 2 Gy (240 kV X-ray, 1 Gy/min) or receiving sham irradiation, respectively.

Quantification of the 2,7-dichlorfluorescein (DCF, H_2_DCF oxidized by ROS fluorescent signal was performed immediately after irradiation using an imaging flow cytometer (Amnis ImageStreamX Mk II, Luminex corp., Austin, TX, USA). Fluorophore excitation was performed using a 488 nm wavelength laser and emission spectra were recorded in Channel 02, no notch filters (no adjacent lasers) active, displaying 505–566 nm. Fluorescence intensity of Channel 02 (DCF) was used to quantify ROS.

### DNA damage γ-H2AX + 53BP1 focus assay

Blood samples were taken from a healthy volunteer as indicated above. For analysis of DNA double-strand break foci we used the γ-H2AX + 53BP1 focus assay as previously described (Eberlein et al. 2015; Lamkowski et al. 2014). Briefly, PBMC were isolated by Ficoll-paque (GE healthcare) density centrifugation at 1800×*g* for 20 min at room temperature. Cells were washed twice with PBS (pH 7,4; without Mg^2+^/Ca^2+^) and fixed in ice-cold 70% ethanol. For immunofluorescence staining (IF) we applied primary mouse anti-phospho(Ser139)-Histone H2A.X (Merck Chemicals; diluted 1:500) and rabbit anti-53BP1 (Novus Bio; diluted 1:500) antibodies and detected them with secondary goat anti-mouse Alexa-488 (Mobitec) and donkey anti-rabbit Cy3-labeled antibodies (Dianova) both at 1:1.000 dilution. Colocalization of γ-H2AX and 53BP1 foci was considered as indicative for DSB formation. Leukocyte nuclei (*n* = 100 per sample) were analyzed by an experienced investigator (H.S.) by manual focus enumeration using a Zeiss Axioimager 2i epifluorescence microscope equipped with a 63 × Planapochromat lens and red/green double bandpass filter (Chroma). Overlapping or deformed nuclei were excluded from the analysis. Only colocalizing γ-H2AX + 53BP1-positive foci were considered for enumeration. Images were recorded using the ISIS fluorescence imaging system (MetaSystems, Germany).

### Cytokinesis-block micronucleus assay (CBMN)

Cell suspensions were prepared by adding either whole blood or CPT™-isolated PBMC to RPMI-1640 supplemented with 200 mM L-glutamine (Gibco-BRL, Germany), antibiotics (100 IU mL-1 penicillin, 100 µg mL-1 streptomycine; Sigma-Aldrich, Germany) and 20% fetal calf serum (PAA, Austria) at a ratio of 1:10. Immediately after irradiation, lymphocytes were stimulated by the addition of 100 µL phytohemagglutinin (PHA-M, Gibco-BRL, Germany). Cultures were incubated for 70 h at 37 °C in a 5% CO_2_ atmosphere. Cytochalasin b was added (final concentration 6 µg/mL; Merck, Germany) 23 h after irradiation and culture setting to block cytokinesis. The fixation procedure was performed at room temperature. Cultures were centrifuged (180*g*, 10 min), treated with a cold hypotonic solution (0,75 M KCl, Gibco-BRL, Germany) and immediately centrifuged again (180*g*, 5 min) before fixation with the freshly prepared cold fixative (methanol:acetic acid 4:1; Roth, Germany) diluted 1/1 with Ringer solution (Merck, Germany). The solution has been added by drops while the tube was agitated with a vortex. Afterwards, cells were centrifuged (180*g*, 8 min) and suspended in three changes of freshly prepared cold fixative (methanol:acetic acid 4:1) without Ringer solution.

Slides with binucleated cells (BN) were prepared at room temperature around 21 °C and humidity around 45% by manually dispensing 35 µL of a suitable concentrated cell suspension with a pipette onto clean dry slides. Slides were coded, air-dried and stored at room temperature until fluorescence staining with 1 drop of Vectashield-DAPI (DAPI: 4′,6-diamidino-2-phenylindole; Axxora, Germany). Scoring of MNi in BN was performed in a semi-automated manner using a complete Metafer4 scanning system with the MSearch/MNScore software (MetaSystems, Germany) (Willems et al. 2010; Thierens et al. 2014). After slide scanning, the software displays the total number of BN is displayed within a cell gallery, the number of MN which has been automatically detected in each BN as well as the MN distribution histogram. In the semi-auto scoring mode all MN-containing BN have been visually inspected in the screen gallery by a trained scorer to correct the number of automatically detected MN if the scorer recognized more or less MN in the respective BN. For each treatment group at least 1000 BN cells from at least three independent experiments were evaluated.

The CBMN was also performed using PBMC to exclude a potential effect of erythrocytes and granulocytes on IR-induced MNi formation in the w/wo CDDO-Me in the experiments using whole blood (conditions using PBMC: 2 h/6 h pre-treatment with 50 nM CDDO-Me).

### Viability assay

ATP content indicating viable cells was determined using the Cell Titer Glo™ Assay (Promega, USA) according to the manufacturers´ instruction. In short, PBMC were seeded in 96 well plates with 40.000 cells per well. Cells were pre-treated with CDDO-Me or the solvent control (6 h), irradiated (2 Gy) or left outside the cabinet in parallel (0 Gy) and after 24 h the Cell Titer Glo™ reagent was added. After stabilization of the signal luminescence was recorded with a Tecan Infinity M200 luminometer (Tecan, Crailsheim, Germany).

### Analysis of apoptosis

For real-time quantification of apoptosis Annexin V-positive cells were measured in the IncuCyte system (IncuCyte S3, Essen BioScience, USA). PBMC (10,000 cells/96 well) were pre-incubated with CDDO-Me (20 nM, 50 nM) or the solvent control DMSO for 6 h and subsequently stained with Annexin V green reagent (Essen BioScience) according to the manufacturers´ protocol. For sham irradiated controls and after acute 2 Gy irradiation every 2 h a picture was taken to monitor apoptosis induction over 48 h. The integrated IncuCyte software was used to count Annexin V-positive cells which were normalized to PBMC confluence.

### Statistical methods

Differences among treatment groups of the γ-H2AX + 53BP1 focus assay were tested for significance with ANOVA followed by the Bonferroni multiple comparisons post-hoc test using SigmaPlot 14.0 (Systat Software, Erkrath, Germany).

For the CBMN assay statistical values were calculated and graphs were plotted using Sigma Plot 13.0 (Systat Software, Erkrath, Germany). The nuclear division index (NDI) is a marker of cell cycle progression of the lymphocytes after mitogenic stimulation which is considered a measure of general cytotoxicity (Fenech 2000). The lowest NDI value is 1.0 if all of the viable cells have failed to divide during the cytokinesis-block period and therefore will be still mononucleated. If all viable cells complete one division they will be all binucleated, the NDI value is 2.0. The NDI has been calculated using the formula NDI = (*M*_1_ + 2*M*_2_)/*N*. *M*_1_ to *M*_2_ represent the number of cells with one or two nuclei, and *N* is the total number of viable cells scored. Cells with 3 or more nuclei have not been observed.

## Results

### CDDO-Me treatment increases antioxidative protein levels in PBMC

Immunoblotting was used to confirm that low nanomolar concentrations of CDDO-Me activate the expression of antioxidative Nrf2/ARE downstream targets. After 6 h of pre-incubation with 50 nM CDDO-Me, human PBMC displayed upregulated levels of HO-1, SOD2 and NQO1 compared to DMSO-treated PBMC as control (Fig. 1b). Administration of 20 nM CDDO-Me for 6 h still markedly increased cellular concentrations of HO-1 and SOD2 (Fig. 1b).

### Nanomolar CDDO-Me enhances ROS activity

**Fig. 2:**
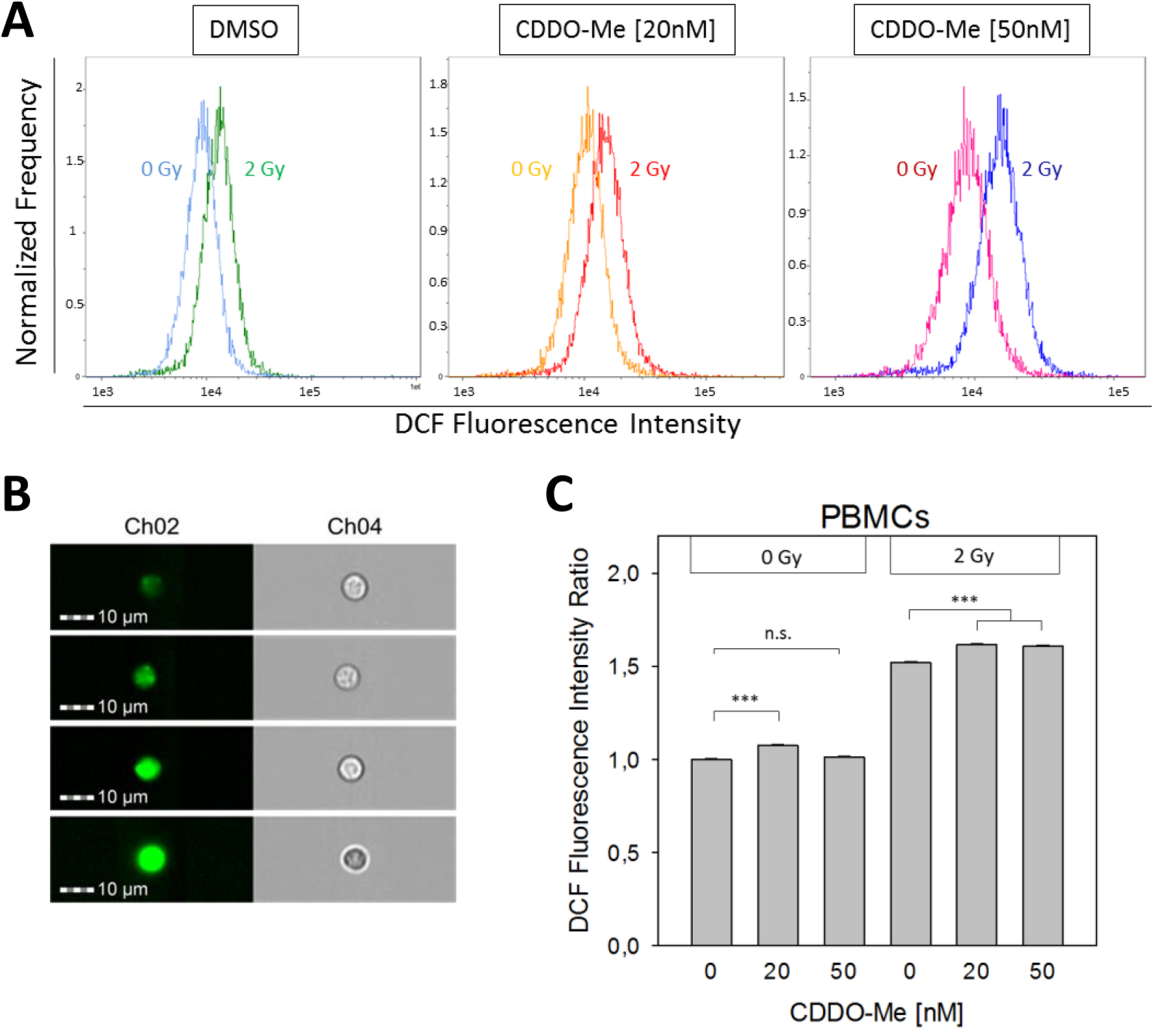
Cellular ROS activity was measured by ImageStream™ flow cytometry using the fluorogenic dye DCF. **a** PBMCs were treated with 0 nM (DMSO), 20 nM or 50 nM CDDO-Me for 5.5 h before DCFDA-labeling for 0.5 h and subsequent irradiation (sham versus 2 Gy). ROS induction by 2 Gy significantly increased DCF fluorescence intensity. **b** Representative pictures of PBMCs show differential DCF fluorescence intensity levels (Ch2) and corresponding brightfield microscopy (Ch4). **c** ROS activity appeared to be significantly elevated by 20 nM CDDO-Me treatment under both, irradiation and sham conditions. Statistics: one way analysis of variance, Bonferroni post-hoc test, ***p < 0.001;* n.s*. not significant; *n* > 11.000 (error bars: SEM)

We investigated ROS generation within PBMC by imaging flow cytometry using the fluorochrome DCF as ROS-indicator. DCF fluorescence signal intensity was significantly increased in all treatment groups when irradiating H_2_DCF-labeled PBMC with 2 Gy (Fig. 2a, b). Single treatment of PBMC with 20 nM (but not 50 nM) CDDO-Me for 5.5 h resulted in a significantly elevated ROS-induced oxidation of intracellular H_2_DCF to fluorogenic DCF during the following 0.5 h (Fig. 2c). ROS-indicative DCF levels remained to be significantly increased in the presence of 20 nM (and 50 nM) CDDO-Me after applying 2 Gy.

### CDDO-Me treatment fails to alter radiation-induced γH2AX + 53BP1 DSB foci in PBMC

**Fig. 3:**
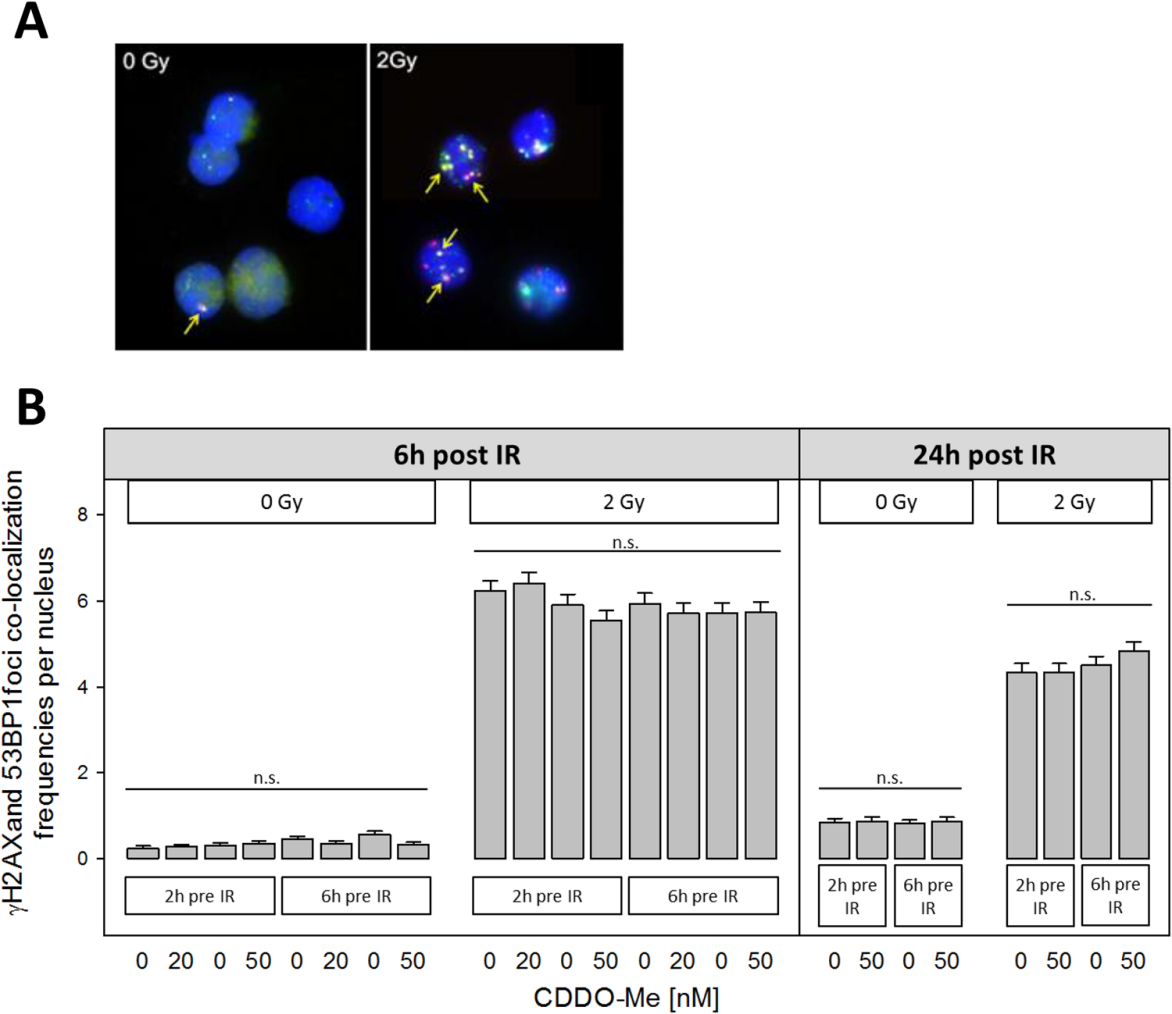
**a** IF staining for DSB-indicating γ-H2AX (green) + 53BP1 (red) foci. Colocalizing colors (reddish/yellow) indicate DSB foci (examples arrowed) in PBMC nuclei (blue) 6 h after 2 Gy irradiation versus control (0 Gy). **b** γ-H2AX + 53BP1 DSB foci frequencies in human PBMCs after 2 or 6 h pre-incubation with nanomolar concentrations of CDDO-Me showed no significant changes when fixing cells 6 h or 24 h post IR. DMSO served as control (0 nM CDDO-Me). Statistics: one way analysis of variance, Bonferroni post-hoc test, *n.s*. not significant for *p* > 0.05; *n* = 100 cells per sample (error bars: SE)

Radiation exposure resulted in a significantly elevated frequency of radiation-induced γ-H2AX + 53BP1 DSB foci in PBMC (Fig. 3a). One Way Analysis of Variance (ANOVA) revealed insignificant differences of γ-H2AX + 53BP1 DSB foci values for all CDDO-Me treated samples and the respective DMSO controls. Thus, using the γ-H2AX + 53BP1 DSB assay, 20 nM or 50 nM CDDO-Me treatment for 2 h or 6 h before irradiation failed to alter the DSB frequency relative to control when analyzing isolated or in-blood irradiated PBMC 6 h as well as 24 h after irradiation, suggesting that CDDO-Me treatment exerts no protective effect with regard to IR-induced DSB formation (Fig. 3b).

### Similar micronuclei formation in binuclear PBMC upon X-irradiation with or without CDDO-Me treatment

**Fig. 4:**
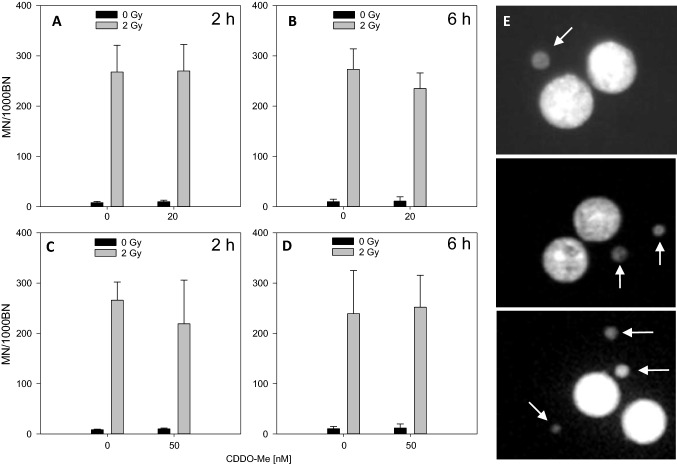
MNi frequencies in PBMC after pre-treatment with CDDO-Me (**a** 2 h/20 nM; **b** 6 h/20 nM; **c** 2 h/50 nM; **d** 6 h/50 nM). Error bars: standard deviations of four independent experiments. DMSO served as control (0 nm CDDO-Me). **e** Representative DAPI-stained binucleated human peripheral blood lymphocyte (harboring one, two and three MNi, arrows) scanned with Metafer4 platform

**Fig. 5:**
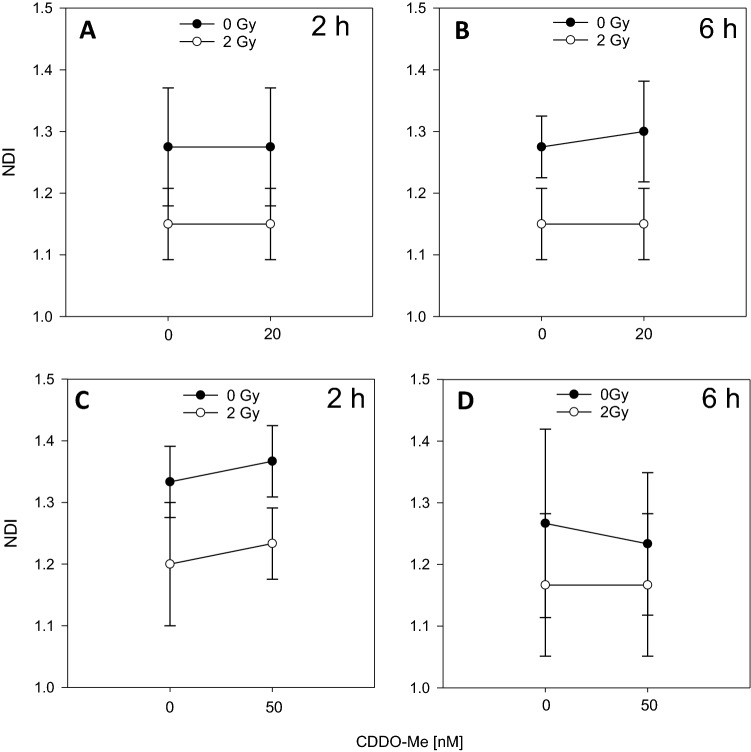
Nuclear division index (NDI) of PBMC after incubation with CDDO-Me (**a** 2 h/20 nM; **b** 6 h/20 nM; **c** 2 h/50 nM; **d** 6 h/50 nM). DMSO served as control (0 nM CDDO-Me). Error bars: standard deviations of four independent experiments

To test further for DNA damage alterations by CDDO-Me, we applied the cytokinesis-block micronucleus assay using PHA-stimulated whole blood cell cultures (Figs. 4, 5) as well as PHA-stimulated PBMC cultures (data not shown). As PHA stimulates human T-lymphocytes the MNi formation is attributed to the activated T cell fraction of whole blood cells or isolated PBMC. The CBMN was performed using PBMC to determine if erythrocytes and granulocytes (present in whole blood cultures) exert an influence on IR-induced MNi formation, potentially modulated by CDDO-Me pre-treatment (conditions tested: 2 and 6 h pre-treatment w/wo 50 nM CDDO-Me). Like in whole blood cultures CDDO-Me pre-treatment did not lead to a decrease of MNi formation in stimulated PMBC (data not shown). Due to the huge amount of peripheral blood to get enough PBMC for MN analysis at all conditions (20 and 50 nm, 2 and 6 h pre-treatment, 0 Gy and 2 Gy, DMSO controls) we used whole blood cultures for the extensive approaches.

Ionizing radiation (2 Gy) induced a significantly increased micronuclei (MNi) frequency (Fig. 4a–d), but a decrease of the nuclear division index (NDI) (Fig. 5a–d) in whole blood cultures as well as in PBMC cultures (data not shown). MNi frequency appeared to be unchanged upon CDDO-Me treatment, indicating that CDDO-Me treatment conditions used for this study exert no genotoxic effects in PBMC. Incubation of human PBMC with nanomolar concentrations of CDDO-Me (20 nM, 50 nM) for 2 or 6 h revealed no increase in MNi frequency of binuclear PBMC. We neither observed a decreased frequency of radiation-induced MNi (Fig. 4) nor an increased NDI (Fig. 5) after CDDO-Me treatment of PBMC for 2 or 6 h before irradiation (20 nM/50 nM) versus DMSO control (0 nM). Thus, using the CBMN assay no decreased frequency of radiation-induced MNi was detected in the presence of CDDO-Me, being in agreement with unaltered DSB foci frequencies noted above.

### PBMC viability and induction of apoptosis is left unchanged by CDDO-Me irrespective of irradiation

**Fig. 6:**
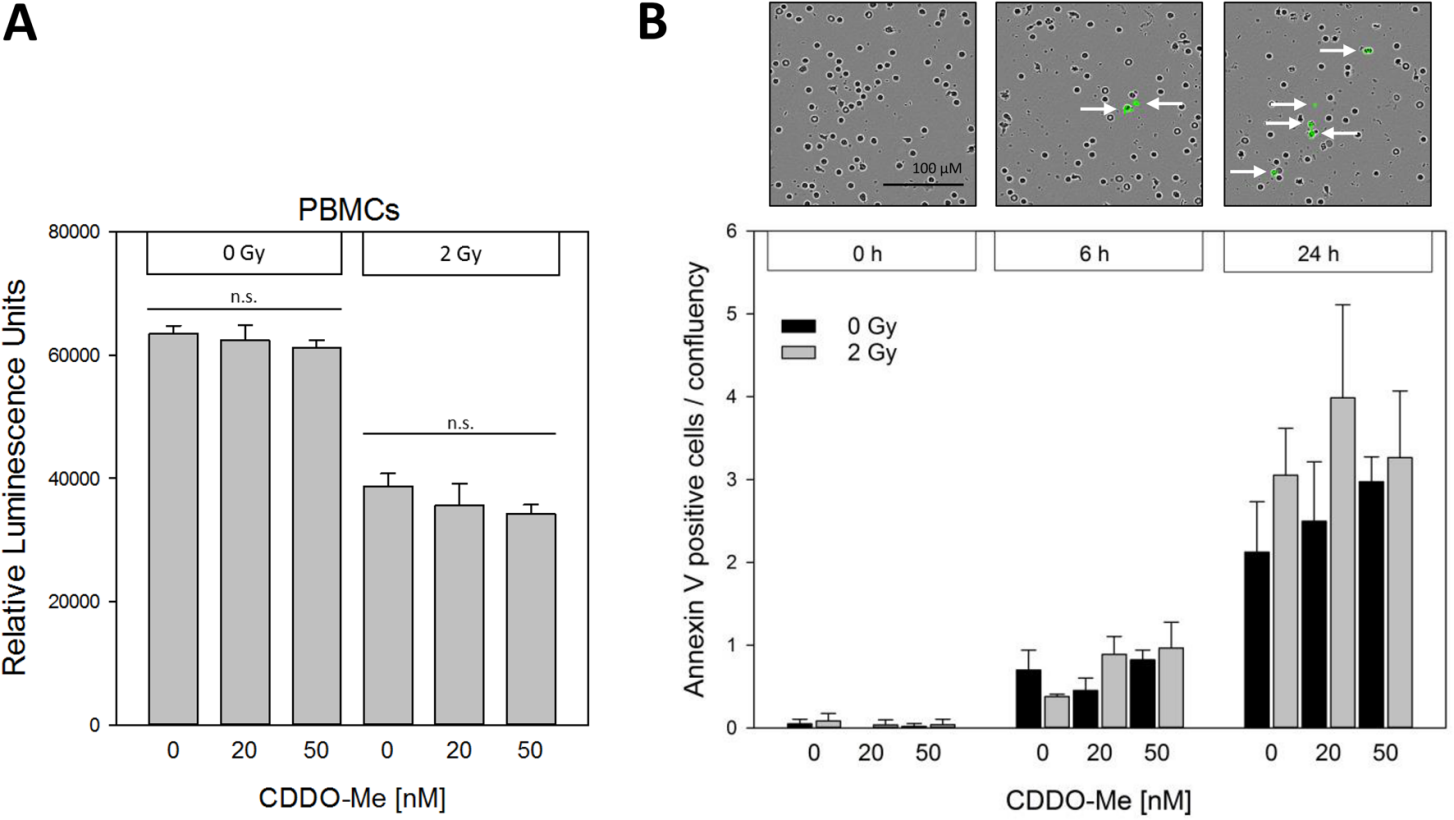
**a** Viability of PBMCs after incubation with CDDO-Me (20 nM/50 nM; administration 6 h before irradiation). Cellular ATP content as a measure of cell viability was determined using the Cell Titer Glo™ Assay. **b** Induction of apoptosis was monitored in the live cell imaging IncuCyte S3 system by quantification of Annexin V-positive cells (green) and subsequent normalization considering confluency. Time-dependent increase of Annexin V-positive cells (green; arrows) is shown in representative pictures. No significant changes were measurable between the treatment groups at the respective incubation time points. Statistics: one way analysis of variance, Bonferroni post-hoc test, *n.s*. not significant for *p* > 0.05; *n* = 3 (error bars: SD)

Viability of PBMC was analyzed via quantification of cellular ATP amounts as representative for the presence of metabolically active cells. Equivalently to our findings regarding DSB repair foci, CDDO-Me treatment displayed no significant impact on PBMC viability. In particular, IR-induced decline of cellular ATP levels was not prevented or even attenuated by the presence of CDDO-Me (Fig. 6a). To further strengthen these findings, we analyzed the induction of apoptosis via live cell imaging by labeling of Annexin V for this purpose. The number of Annexin V-positive cells increased over time. During the time span of 24 h, CDDO-Me again showed neither significant cytoprotective nor radioprotective effects (Fig. 6b).

## Discussion

Previous studies highlighted the potential role of the synthetic triterpenoid CDDO-Me as an effective radioprotective or -mitigative small molecule drug to overcome harmful effects of ionizing radiation for healthy human tissue, mainly via the induction of antioxidative Nrf2/ARE downstream targets. In this study, we confirm that treatment of human PBMC with nanomolar CDDO-Me concentrations is followed by increased cellular levels of antioxidative HO-1, mitochondrial SOD2 and NQO1 within few hours. In parallel, we found, against expectations, ROS activity in PBMC to be enhanced by short-term CDDO-Me pre-treatment. However, the increased ROS levels did not lead to changed DSB levels, as measured by the γ-H2AX + 53BP1 focus assay, which was reflected by the absence of significant changes in apoptosis.

Commonly, biological effects of ionizing radiation are assigned to the generation of various DNA damages such as single-strand breaks (SSB), inter-DNA crosslinks, base lesions or DSB, with the latter representing the most momentous effect due to the initiation of error-prone DNA damage repair (DDR) mechanisms (Goodhead et al. 1993; Mladenova et al. 2016; Lassmann et al. 2010). Any form of IR-induced DNA damage can principally be generated either by direct ionization of DNA molecules or by indirect action with other atoms or molecules via generation of highly reactive molecules (ROS, RNS).

CDDO-Me has been shown to exert beneficial antioxidative effects on normal tissue at low nanomolar concentrations (Liby and Sporn 2012). Several studies demonstrated a CDDO-Me mediated upregulation of antioxidative HO-1 levels in human epithelial cells derived from colon, lung, breast and bronchus as well as in PBMC and neutrophils, which goes in line with our findings (El-Ashmawy et al. 2014; Thimmulappa et al. 2007; Kim et al. 2012; Eskiocak et al. 2010). Direct interaction with Keap1 and the subsequent activation of the Nrf2/ARE pathway are regarded as the key mechanism for the downstream induction of antioxidant proteins and enzymes (Liby and Sporn 2012; Sporn et al. 2011). Accordingly, Eskiocak et al. revealed decreased ROS upon 50 nM CDDO-Me treatment over 16 h in immortalized human colonic epithelial cells (HCECs) (Eskiocak et al. 2010). A further study highlighted attenuated ROS generation by various stimuli when incubating peripheral blood neutrophils with 20 nM CDDO-Im, an imidazole derivative of CDDO for 20 h (Thimmulappa et al. 2007). At first sight, these findings seem to contrast with the elevated ROS activity in PBMC after treatment with 20 nM CDDO-Me for 6 h observed in our study. However, tumor cells often display elevated ROS levels, while the upregulation of Nrf2/ARE defense targets in PBMC may have attenuated the detrimental effects of the increased cellular ROS in these normal cells. Apart from differences regarding the used cellular models or CDDO-derivates, in particular, the markedly longer pre-treatment times applied in other studies may underline the different response of normal PBMC noted here.

ROS are inevitable by-products of aerobic metabolism and generated at the inner mitochondrial membrane during oxidative phosphorylation. The resulting proton gradient finally drives the mitochondrial ATP-synthase. CDDO-Me has been shown to activate multiple signaling pathways, ending up in the long term with increased activities of various ATP-consuming transcription and translation processes (Liby and Sporn 2012). Therefore, CDDO-Me might directly or indirectly activate the mitochondrial respiratory chain and ROS production to maintain cellular ATP- levels in a first phase. Increased ROS activity may partially even trigger the transcription of ARE-regulated genes and the up-regulation of detoxifying enzymes like SOD2 as the main mitochondrial antioxidative defensive mechanism (Storz et al. 2005). While radiation-induced ROS are mediating damage to molecules throughout the cell including nuclear DNA, cell-generated ROS via enhanced mitochondrial metabolism may largely be neutralized by mitochondrial ROS defense mechanisms before impairing DNA integrity within the nucleus. This effect may in part explain the unchanged frequency of DSBs or MNi concomitant to the observed enhancement of ROS levels after CDDO treatment.

In immortalized HCECs CDDO-Me treatment arose as a radioprotective measure by a reduction of IR-related G1 and S/G2 chromosome aberrations and an increase of DNA damage signaling (Kim et al. 2012). Furthermore, CDDO-Me pre-treatment of mice enhanced median survival after 7.5 Gy total body irradiation (TBI) and protected the murine GI tract by the reduction of apoptosis, the preservation of proliferative crypt cells and an increase in DDR efficacy as shown by a reduced frequency of 53BP1-positive crypt cells 5 days after TBI (Kim et al. 2012). Eskiocak et al. revealed radioprotective effects of CDDO-Me, when 18 h pretreated HCECs were exposed to γ-irradiation in that γ-H2AX foci were reduced by 15–30% compared to DMSO control 0.5 h after IR (Eskiocak et al. 2010). However, this and our study employed photon irradiation of different energies and dose rates, which renders the results difficult to compare. This consideration becomes even more evident with regard to likely consequences arising from cell type differences (cycling tumor cells vs G1/G0 PBMC) and the strikingly different time points of analysis after IR (0.5 h vs 6 h and 24 h in our study). Last but not least, the obvious differences within the applied pre-treatment times (18 h vs 2 and 6 h in this study) may also explain the contrasting findings. In conclusion, the increased endogenous ROS activity during the first hours of CDDO-treatment may have nullified the radioprotective effects mediated by up-regulated antioxidative enzymes.

Recently, a first study demonstrated CDDO-Me-related protection of human PBMC against IR (El-Ashmawy et al. 2014) used 18 h pre-treatment with10 nM CDDO-Me prior to 3 Gy γ-radiation exposure at 2.4 Gy/min using a Cs-137 source. Subsequently, an alkaline comet assay was performed to determine DNA damage. After IR, the tail moment appeared to be significantly reduced upon CDDO-Me, which seems to contrast with the findings of the present study. However, since the alkaline comet assay reveals all types of DNA strand breaks in total, including the more frequent IR-induced SSBs, it seems possible that the COMET results of El-Ashmawy et al. (El-Ashmawy et al. 2014) overestimate the amount of dsDNA damage, whereas the methods used in our study directly detect DSBs. In agreement, it has been observed that γ-H2AX DSB foci are the more sensitive measure for dsDNA damage (Yu et al. 2006) and that the neutral COMET assay correlates more significantly with clonogenic survival of irradiated cells (Jayakumar et al. 2012). Furthermore, the longer CDDO-Me incubation period of 18 h of El-Ashmawy et al. (versus 2 and 6 h in our study) along with the shorter post-IR time-points for analysis of 0.5 h (versus 6 h and 24 h in our study) may again represent main factors for the observed differential findings, which will be addressed in future experiments. Finally, the different radiation exposure protocols impede the comparability of the two studies, since the relative biological effectiveness of ionizing radiation crucially depends on the linear energy transfer (662 keV versus 100 keV photons), the dose (3 Gy versus 2 Gy), the dose rate (2.4 Gy/min versus 1.0 Gy/min) and the analyzed biological end points.

To our knowledge, we are the first to combine the sensitive γ-H2AX + 53BP1 focus assay with a CBMN assay to analyze potential radioprotective effects of low nanomolar CDDO-Me on radiation-induced DSB induction and repair in human PBMC. While the γ-H2AX + 53BP1 co-staining is a highly sensitive method to detect DSBs after their induction (Markova et al. 2011; Lassmann et al. 2010; Eberlein et al. 2015), it also avoids misinterpretation of γ-H2AX foci alone, since γ-H2AX formation may also be induced by non-DSB-inducing treatments like heat or cell cycle drugs (Tu et al. 2013; Takahashi et al. 2008).

The CBMN assay, on the other hand, follows the consequences of DSB induction and failure of repair indicated by chromosomes and fragments after the first nuclear division during mitosis post irradiation. Since common IR-induced G1-specific aberrations detected at metaphase are mostly of the chromosomal type and include a high frequency of dicentric chromosomes and acentric fragments (Moore and Bender 1993), we applied the CBMN assay as a screening assay. Using this assay we observed that CDDO-Me treatment did not influence DSB formation and DSB repair, as both control and treated samples showed similar MNi frequencies w/wo irradiation, results that corroborate our findings of similar DSB induction in the presence or absence of CDDO-Me.

## Conclusions

Considering our results, short-term pre-treatment with CDDO-Me and subsequent irradiation of PBMC displayed neither a significantly altered DSB-formation nor induction of apoptosis. We suggest that the lack of measurable radioprotective effects upon short-term CDDO-Me treatment may result from the balance between the protective up-regulation of antioxidant proteins HO-1, NQO1 and SOD-2 and the potentially detrimental consequences of mitochondrial ROS production due to an enhanced metabolic rate. Therefore, future investigations shall address DSB damage and its response in CDDO-Me-treated PBMC and their cellular metabolic activity after extended CDDO-Me treatment times. Nevertheless, the presence of antioxidative proteins may make a decisive difference in altering the intracellular long-term effects of ROS generated by stress-induced hyper-metabolism during a DNA damage response, which could explain the observed radioprotective characteristics of CDDO-Me in the long term.

## References

[CR1] Alqahtani A, Hamid K, Kam A, Wong KH, Abdelhak Z, Razmovski-Naumovski V, Chan K, Li KM, Groundwater PW, Li GQ (2013). The pentacyclic triterpenoids in herbal medicines and their pharmacological activities in diabetes and diabetic complications. Curr Med Chem.

[CR2] Azzam EI, Jay-Gerin JP, Pain D (2012). Ionizing radiation-induced metabolic oxidative stress and prolonged cell injury. Cancer Lett.

[CR3] Bhattacharjee Y (2011). Devastation in Japan. Candidate radiation drugs inch forward. Science (New York, NY).

[CR4] Dinkova-Kostova AT, Talalay P (2010). NAD(P)H:quinone acceptor oxidoreductase 1 (NQO1), a multifunctional antioxidant enzyme and exceptionally versatile cytoprotector. Arch Biochem Biophys.

[CR5] Durante M, Cucinotta FA (2008). Heavy ion carcinogenesis and human space exploration. Nat Rev Cancer.

[CR6] Eberlein U, Peper M, Fernandez M, Lassmann M, Scherthan H (2015). Calibration of the gamma-H2AX DNA double strand break focus assay for internal radiation exposure of blood lymphocytes. PLoS ONE.

[CR7] El-Ashmawy M, Delgado O, Cardentey A, Wright WE, Shay JW (2014). CDDO-Me protects normal lung and breast epithelial cells but not cancer cells from radiation. PLoS ONE.

[CR8] Eskiocak U, Kim SB, Roig AI, Kitten E, Batten K, Cornelius C, Zou YS, Wright WE, Shay JW (2010). CDDO-Me protects against space radiation-induced transformation of human colon epithelial cells. Radiat Res.

[CR9] Fenech M (2000). The in vitro micronucleus technique. Mutat Res.

[CR10] Goodhead DT, Thacker J, Cox R (1993). Weiss lecture. Effects of radiations of different qualities on cells: molecular mechanisms of damage and repair. Int J Radiat Biol.

[CR11] Honda T, Rounds BV, Bore L, Finlay HJ, Favaloro FG, Suh N, Wang Y, Sporn MB, Gribble GW (2000). Synthetic oleanane and ursane triterpenoids with modified rings A and C: a series of highly active inhibitors of nitric oxide production in mouse macrophages. J Med Chem.

[CR12] Jayakumar S, Bhilwade HN, Pandey BN, Sandur SK, Chaubey RC (2012). The potential value of the neutral comet assay and the expression of genes associated with DNA damage in assessing the radiosensitivity of tumor cells. Mutat Res.

[CR13] Kim SB, Pandita RK, Eskiocak U, Ly P, Kaisani A, Kumar R, Cornelius C, Wright WE, Pandita TK, Shay JW (2012). Targeting of Nrf2 induces DNA damage signaling and protects colonic epithelial cells from ionizing radiation. Proc Natl Acad Sci USA.

[CR14] Kim SB, Ly P, Kaisani A, Zhang L, Wright WE, Shay JW (2013). Mitigation of radiation-induced damage by targeting EGFR in noncancerous human epithelial cells. Radiat Res.

[CR15] Lamkowski A, Forcheron F, Agay D, Ahmed EA, Drouet M, Meineke V, Scherthan H (2014). DNA damage focus analysis in blood samples of minipigs reveals acute partial body irradiation. PLoS ONE.

[CR16] Lassmann M, Hanscheid H, Gassen D, Biko J, Meineke V, Reiners C, Scherthan H (2010). In vivo formation of gamma-H2AX and 53BP1 DNA repair foci in blood cells after radioiodine therapy of differentiated thyroid cancer. J Nucl Med.

[CR17] Lebovitz RM, Zhang H, Vogel H, Cartwright J, Dionne L, Lu N, Huang S, Matzuk MM (1996). Neurodegeneration, myocardial injury, and perinatal death in mitochondrial superoxide dismutase-deficient mice. Proc Natl Acad Sci USA.

[CR18] Liby KT, Sporn MB (2012). Synthetic oleanane triterpenoids: multifunctional drugs with a broad range of applications for prevention and treatment of chronic disease. Pharmacol Rev.

[CR19] Maier P, Hartmann L, Wenz F, Herskind C (2016). Cellular pathways in response to ionizing radiation and their targetability for tumor radiosensitization. Int J Mol Sci.

[CR20] Markova E, Torudd J, Belyaev I (2011). Long time persistence of residual 53BP1/gamma-H2AX foci in human lymphocytes in relationship to apoptosis, chromatin condensation and biological dosimetry. Int J Radiat Biol.

[CR21] Mirman Z, de Lange T (2020). 53BP1: a DSB escort. Genes Dev.

[CR22] Mladenova V, Mladenov E, Iliakis G (2016). Novel biological approaches for testing the contributions of single DSBs and DSB clusters to the biological effects of high LET radiation. Front Oncol.

[CR23] Moore RC, Bender MA (1993). Chromosomal aberration types in cells at the second division after irradiation in G1 or G2. Int J Radiat Biol.

[CR24] Petronelli A, Pannitteri G, Testa U (2009). Triterpenoids as new promising anticancer drugs. Anticancer Drugs.

[CR25] Riley PA (1994). Free radicals in biology: oxidative stress and the effects of ionizing radiation. Int J Radiat Biol.

[CR26] Rogakou EP, Pilch DR, Orr AH, Ivanova VS, Bonner WM (1998). DNA double-stranded breaks induce histone H2AX phosphorylation on serine 139. J Biol Chem.

[CR27] Rogakou EP, Boon C, Redon C, Bonner WM (1999). Megabase chromatin domains involved in DNA double-strand breaks in vivo. J Cell Biol.

[CR28] Ryu SY, Oak MH, Yoon SK, Cho DI, Yoo GS, Kim TS, Kim KM (2000). Anti-allergic and anti-inflammatory triterpenes from the herb of *Prunella vulgaris*. Planta Med.

[CR29] Shanmugam MK, Nguyen AH, Kumar AP, Tan BK, Sethi G (2012). Targeted inhibition of tumor proliferation, survival, and metastasis by pentacyclic triterpenoids: potential role in prevention and therapy of cancer. Cancer Lett.

[CR30] Shanmugam MK, Dai X, Kumar AP, Tan BK, Sethi G, Bishayee A (2014). Oleanolic acid and its synthetic derivatives for the prevention and therapy of cancer: preclinical and clinical evidence. Cancer Lett.

[CR31] Shay JW, Cucinotta FA, Sulzman FM, Coleman CN, Minna JD (2011). From mice and men to earth and space: joint NASA-NCI workshop on lung cancer risk resulting from space and terrestrial radiation. Can Res.

[CR32] Son Y, Lee JH, Chung HT, Pae HO (2013). Therapeutic roles of heme oxygenase-1 in metabolic diseases: curcumin and resveratrol analogues as possible inducers of heme oxygenase-1. Oxid Med Cell Longev.

[CR33] Sporn MB, Liby KT, Yore MM, Fu L, Lopchuk JM, Gribble GW (2011). New synthetic triterpenoids: potent agents for prevention and treatment of tissue injury caused by inflammatory and oxidative stress. J Nat Prod.

[CR34] Storz P, Doppler H, Toker A (2005). Protein kinase D mediates mitochondrion-to-nucleus signaling and detoxification from mitochondrial reactive oxygen species. Mol Cell Biol.

[CR35] Suh N, Wang Y, Honda T, Gribble GW, Dmitrovsky E, Hickey WF, Maue RA, Place AE, Porter DM, Spinella MJ, Williams CR, Wu G, Dannenberg AJ, Flanders KC, Letterio JJ, Mangelsdorf DJ, Nathan CF, Nguyen L, Porter WW, Ren RF, Roberts AB, Roche NS, Subbaramaiah K, Sporn MB (1999). A novel synthetic oleanane triterpenoid, 2-cyano-3,12-dioxoolean-1,9-dien-28-oic acid, with potent differentiating, antiproliferative, and anti-inflammatory activity. Can Res.

[CR36] Suman S, Jain S, Chandna S (2013). Recent patents in the field of radioprotector development: opportunities and challenges. Recent Pat Biotechnol.

[CR37] Takahashi A, Mori E, Somakos GI, Ohnishi K, Ohnishi T (2008). Heat induces γH2AX foci formation in mammalian cells. Mutat Res Genet Toxicol Environ Mutagen.

[CR38] Thierens H, Vral A, Vandevoorde C, Vandersickel V, de Gelder V, Romm H, Oestreicher U, Rothkamm K, Barnard S, Ainsbury E, Sommer S, Beinke C, Wojcik A (2014). Is a semi-automated approach indicated in the application of the automated micronucleus assay for triage purposes?. Radiat Prot Dosimetry.

[CR39] Thimmulappa RK, Fuchs RJ, Malhotra D, Scollick C, Traore K, Bream JH, Trush MA, Liby KT, Sporn MB, Kensler TW, Biswal S (2007). Preclinical evaluation of targeting the Nrf2 pathway by triterpenoids (CDDO-Im and CDDO-Me) for protection from LPS-induced inflammatory response and reactive oxygen species in human peripheral blood mononuclear cells and neutrophils. Antioxid Redox Signal.

[CR40] Tu WZ, Li B, Huang B, Wang Y, Liu XD, Guan H, Zhang SM, Tang Y, Rang WQ, Zhou PK (2013). gammaH2AX foci formation in the absence of DNA damage: mitotic H2AX phosphorylation is mediated by the DNA-PKcs/CHK2 pathway. FEBS Lett.

[CR41] Vral A, Fenech M, Thierens H (2011). The micronucleus assay as a biological dosimeter of in vivo ionising radiation exposure. Mutagenesis.

[CR42] Wang YY, Yang YX, Zhe H, He ZX, Zhou SF (2014). Bardoxolone methyl (CDDO-Me) as a therapeutic agent: an update on its pharmacokinetic and pharmacodynamic properties. Drug Des Dev Ther.

[CR43] Wang YY, Zhe H, Zhao R (2014). Preclinical evidences toward the use of triterpenoid CDDO-Me for solid cancer prevention and treatment. Mol Cancer.

[CR44] Wang YY, Yang YX, Zhao R, Pan ST, Zhe H, He ZX, Duan W, Zhang X, Yang T, Qiu JX, Zhou SF (2015). Bardoxolone methyl induces apoptosis and autophagy and inhibits epithelial-to-mesenchymal transition and stemness in esophageal squamous cancer cells. Drug Des Dev Ther.

[CR45] Willems P, August L, Slabbert J, Romm H, Oestreicher U, Thierens H, Vral A (2010). Automated micronucleus (MN) scoring for population triage in case of large scale radiation events. Int J Radiat Biol.

[CR46] Yu Y, Zhu W, Diao H, Zhou C, Chen FF, Yang J (2006). A comparative study of using comet assay and gammaH2AX foci formation in the detection of *N*-methyl-*N*'-nitro-*N*-nitrosoguanidine-induced DNA damage. Toxicol In Vitro Int J Publ Assoc BIBRA.

